# Associations of the Expression Levels and Risk Variants of *CDKN2B‐AS1* Long Noncoding RNA With the Susceptibility and Progression of Prostate Cancer

**DOI:** 10.1111/jcmm.70264

**Published:** 2024-12-04

**Authors:** Min‐Che Tung, Chia‐Yen Lin, Yu‐Ching Wen, Lun‐Ching Chang, Shun‐Fa Yang, Ming‐Hsien Chien

**Affiliations:** ^1^ Division of Urology, Department of Surgery Tungs' Taichung Metro Harbor Hospital Taichung Taiwan; ^2^ Graduate Institute of Clinical Medicine, College of Medicine Taipei Medical University Taipei Taiwan; ^3^ Division of Urology, Department of Surgery Taichung Veterans General Hospital Taichung Taiwan; ^4^ School of Medicine Chung Shan Medical University Taichung Taiwan; ^5^ School of Medicine National Yang Ming Chiao Tung University Taipei Taiwan; ^6^ Department of Urology, Wan Fang Hospital Taipei Medical University Taipei Taiwan; ^7^ Department of Urology, School of Medicine, College of Medicine and TMU Research Center of Urology and Kidney (TMU‐RCUK) Taipei Medical University Taipei Taiwan; ^8^ Department of Mathematical Sciences Florida Atlantic University Florida USA; ^9^ Institute of Medicine Chung Shan Medical University Taichung Taiwan; ^10^ Department of Medical Research Chung Shan Medical University Hospital Taichung Taiwan; ^11^ Pulmonary Research Center, Wan Fang Hospital Taipei Medical University Taipei Taiwan; ^12^ Traditional Herbal Medicine Research Center Taipei Medical University Hospital Taipei Taiwan; ^13^ TMU Research Center of Cancer Translational Medicine Taipei Medical University Taipei Taiwan

**Keywords:** cancer progression, cancer susceptibility, cyclin‐dependent kinase inhibitor 2B antisense RNA 1, long noncoding RNA, prostate cancer, single‐nucleotide polymorphism

## Abstract

Genetic variants of deregulated long noncoding RNAs (lncRNAs) have been implicated in tumorigenesis, cancer progression and cancer recurrence. Single‐nucleotide polymorphisms (SNPs) of the lncRNA cyclin‐dependent kinase inhibitor 2B antisense RNA 1 (*CDKN2B‐AS1*) have been associated with the risk and progression of various cancers; however, their role in prostate cancer (PCa) remains underexplored. In this case–control study, we investigated the associations of *CDKN2B‐AS1* expression levels and variants with PCa risk and progression. For this, five SNPs of *CDKN2B‐AS1*—rs564398, rs1333048, rs1537373, rs2151280 and rs8181047—were genotyped using a TaqMan allelic discrimination assay; data were collected from 695 patients with PCa and 695 healthy controls. Our findings revealed that, under a dominant model, patients with PCa carrying at least one minor C allele of rs1333048 exhibited an increased risk of developing tumours with high Gleason grades; this risk was particularly high in patients without biochemical recurrence. Data from the Genotype‐Tissue Expression database indicated upregulated *CDKN2B‐AS1* expression in the prostates of individuals carrying the polymorphic C allele of rs1333048. Genotype screening of rs1333048 in PCa cell lines showed that cells with at least one minor C allele had higher *CDKN2B‐AS1* levels than those with the AA genotype. Furthermore, data from The Cancer Genome Atlas indicated that higher *CDKN2B‐AS1* levels in PCa tissues were correlated with larger tumour sizes (T3 + T4), more lymph node metastasis (N1), higher Gleason scores and shorter progression‐free survival. In conclusion, the polymorphic variants of *CDKN2B‐AS1* at rs1333048 may modulate *CDKN2B‐AS1* expression, thus accelerating PCa progression.

## 1 Introduction

Prostate cancer (PCa) is a heterogeneous malignancy characterised by a high prevalence among men in Western countries [1] and in developed countries such as Taiwan [2]. When PCa is diagnosed at an early, localised stage, it can be effectively managed through radical prostatectomy (RP) or radiotherapy (either external beam or brachytherapy), achieving a remarkable 5‐year survival rate of approximately 100% [3]. By contrast, when PCa is diagnosed at a late, metastatic stage, androgen‐deprivation therapy (ADT) is initially effective in most patients. However, many patients eventually have biochemical recurrence (BCR), resulting in a 5‐year survival rate of only 29.3% [4, 5]. Therefore, effective biomarkers are urgently needed for early diagnosis of PCa. Currently, prostate‐specific antigen (PSA) is the most commonly used biomarker for predicting PCa; however, the predictive accuracy of PSA remains limited. Although elevated serum PSA levels are commonly associated with PCa, PSA levels may be elevated also in benign diseases such as prostatic hyperplasia and prostatitis [6]. Moreover, the reliability of PSA as a biomarker diminishes in the advanced stages of PCa [7]. Early diagnosis of PCa is crucial for preventing metastasis and optimising treatment outcomes. Given the limited sensitivity and specificity of PSA, novel and effective predictive biomarkers are urgently required.

Genetic alterations play pivotal roles in the development and progression of PCa [8]. Single‐nucleotide polymorphisms (SNPs), defined as variations in DNA base pairs that occur in > 1% of the population, have been associated with cancer risk, cancer progression and treatment response [9, 10, 11]. For example, the rs1805087 A/G SNP of methionine synthase is associated with an increased risk of PCa [12]. Similarly, the rs145204276 SNP of the growth arrest‐specific 5 gene is associated with an increased risk of PCa metastasis [13]. Finally, the rs11549465 SNP of the hypoxia‐inducible factor (HIF)‐1α gene is associated with an elevated risk of distant metastasis and an increased level of resistance to ADT [14]. Many SNPs identified through genome‐wide association studies are associated with cancer and long noncoding RNAs (lncRNAs) [15]. LncRNAs, a class of noncoding RNAs longer than 200 bp, function as oncogenes or tumour suppressors, playing key roles in various aspects of tumour progression—for example, uncontrolled proliferation, therapeutic resistance and metastasis [16]. SNP mutations may modulate the function of lncRNAs by altering their transcriptional regulatory regions or modifying their secondary structures, thereby affecting microRNA (miRNA) binding sites and influencing lncRNA expression [17].

The lncRNA cyclin‐dependent kinase inhibitor 2B antisense RNA 1 (*CDKN2B‐AS1*), also known as antisense noncoding RNA at the INK4 locus, is transcribed from a gene located within a genomic hotspot on chromosome 9p21.3, a region coding multiple disease‐related SNPs [18]. Genome‐wide association studies have highlighted this locus as a common genetic site associated with susceptibility to various diseases, including cancer [19]. For instance, *CDKN2B‐AS1* SNPs have been associated with large tumour sizes (e.g., rs11333048 in thyroid and oral cancers) [20, 21] as well as advanced TNM stages and high metastasis risks (e.g., rs3217992 in osteosarcoma) [22]. Furthermore, the *CDKN2B‐AS1* SNPs rs1333049 and rs2383207 have been associated with overall survival in patients with head and neck cancer and breast cancer, respectively [23, 24]. Zhao et al. reported that the overexpression of *CDKN2B‐AS1* leads to increased proliferation and migration of PCa cells through the regulation of the let‐7a miRNA/transforming growth factor‐β1(TGF‐β1)/Smad pathway [25]. Despite investigations into the functional roles of *CDKN2B‐AS1* in PCa, the clinical implications of *CDKN2B‐AS1* expression levels and variants in the context of PCa remain unclear. Thus, we conducted the present case–control study to investigate the associations of the expression levels and variants of *CDKN2B‐AS1* with the risk and progression of PCa in the Taiwanese population.

## 2 Materials and Methods

### 2.1 Study Cohorts

This retrospective study included two cohorts: 695 Taiwanese patients with PCa and 695 matched healthy controls (men). All individuals had the same ethnic background and resided in similar geographical regions. The patients with PCa underwent robot‐assisted RP after histological confirmation of the diagnosis at Taichung Veterans General Hospital (Taichung, Taiwan) between 2012 and 2018. At the time of PCa diagnosis, venous blood samples were collected from all patients; in addition, information on demographic characteristics and medical history was collected. The following data were extracted from the patients' medical records: PSA levels, pathological Gleason grades, clinical and pathological T (tumour) and N (node) stages, cancer cell invasion (seminal vesicle and lymphatic channels or blood vessels) and D'Amico classification. BCR in the recruited PCa patients was defined as the detection of two consecutive PSA measurements, each surpassing 0.2 ng/mL. This threshold served as an indicator of potential cancer recurrence after initial treatment. Written informed consent was obtained from all participants before the initiation of this study. The study protocol was approved by the Institutional Review Board of Taichung Veterans General Hospital, Taiwan (permit number: CE19062A‐2).

### 2.2 Genomic DNA Isolation

Whole‐blood samples were aseptically collected from the participants through venipuncture and preserved in ethylenediaminetetraacetic acid‐coated tubes for DNA isolation. Genomic DNA was isolated by using the QIAamp DNA Blood Mini Kit (Qiagen, Valencia, CA, USA). DNA quality was evaluated using the Nanodrop‐2000 spectrophotometer (Thermo Fisher Scientific, Waltham, MA, USA). The final DNA preparations were stored at −20°C for subsequent real‐time polymerase chain reaction (PCR) analysis.

### 2.3 Selection and Determination of *CDKN2B‐* *AS1* SNPs

We selected five *CDKN2B‐AS1* SNPs that are significantly associated with cancer risk or traits such as tumour size across cancers (e.g., lung cancer, thyroid cancer, head and neck cancer, melanoma and pancreatic cancer) [26, 27]: rs564398 (T/C), rs1333048 (A/C), rs1537373 (G/T), rs2151280 (A/G) and rs8181047 (G/A). Notably, most of these SNPs have not been previously investigated in the context of PCa. Allelic discrimination of the *CDKN2B‐AS1* SNPs rs564398 (assay ID: C_2618017_10), rs1333048 (assay ID: C_1754667_10), rs1537373 (assay ID: C_8766806_20), rs2151280 (assay ID: C_11841814_10) and rs8181047 (assay ID: C_30315510_10) was performed using a TaqMan SNP Genotyping Assay by using the ABI StepOnePlus Real‐Time PCR System (Applied Biosystems, Foster City, CA, USA). Allelic variants at the indicated loci were analysed using the SDS software (version 3.0; Applied Biosystems).

### 2.4 PCa Cell Lines and Culture

The human PCa cell lines 22Rv1, DU145, PC3 and PC3‐M were sourced from the American Type Culture Collection (Manassas, VA, USA). DU145 cells were grown in Dulbecco's modified Eagle medium (DMEM, Life Technologies, Grand Island, NY, USA); PC3 and PC3‐M cells in minimum essential medium (MEM, Life Technologies); and 22Rv1 cells in RPMI‐1640 medium (Life Technologies). Each culture medium was supplemented with 10% foetal bovine serum and 1% penicillin–streptomycin–glutamine (Life Technologies). Cells were incubated at 37°C in an atmosphere of 5% CO₂ and 95% air.

### 2.5 Extraction of RNA and Reverse‐Transcriptase Quantitative Polymerase Chain Reaction (RT‐qPCR)

Total RNA was extracted from PCa cell lines using TRIzol reagent. Reverse transcription was carried out with the iScript cDNA Synthesis Kit (Bio‐Rad, Hercules, CA, USA). Quantitative PCR (qPCR) was then conducted using TOOLS 2X SYBR qPCR Mix (BIOTOOLS, Taipei, Taiwan) and *CDKN2B‐AS1*‐specific primers according to the manufacturer's instructions. *CDKN2B‐AS1* expression levels were normalised to *GAPDH* as the internal control. The primer sequences were as follows: CDKN2B‐AS1_F: GAC TTC TGT TTT CTG GCC ACC and CDKN2B‐AS1_R: TCG GGA AAG GAT TCC AGC AC.

### 2.6 Bioinformatic Analysis

RNA sequencing data and clinical data of patients with prostate adenocarcinoma (PRAD) were obtained from The Cancer Genome Atlas (TCGA) through the University of California, Santa Cruz Xena tool (https://xena.ucsc.edu/). The Wilcoxon test was used to evaluate and compare *CDKN2B‐AS1* expression between tumour and normal tissues and across tumours with varying pathological T and N stages and Gleason scores. To analyse progression‐free survival (PFS), patients with PRAD were stratified into high‐ and low‐expression groups by the median expression level of *CDKN2B‐AS1*. Statistical significance was determined using a log‐rank test. To identify *CDKN2B‐AS1*‐associated pathways, a gene set enrichment analysis (GSEA) was performed. Genes were ranked by the coefficients of their correlation with *CDKN2B‐AS1*, and the normalised enrichment score (NES) and false discovery rate (FDR) were calculated using the weighted Kolmogorov–Smirnov test. Pathways with a FDR of < 0.05 were regarded as statistically significant. Furthermore, the correlations of the gene expression level of *CDKN2B‐AS1* with those of E‐cadherin (*CDH1*), tight junction protein 1 (*TJP1*), N‐cadherin (*CDH2*), fibronectin 1 (*FN1*), vimentin (*VIM*), interleukin‐6 (*IL6*), tumour necrosis factor‐α (*TNF*) and interferon‐γ (*IFNG*) in patients with PCa were evaluated using the cBioportal platform (https://www.cbioportal.org//).

### 2.7 Statistical Analysis

The correlations of the frequencies of various *CDKN2B‐AS1* variants with the risk of PCa or related clinicopathological features were investigated using multivariate logistic regression models; corresponding odds ratios (ORs), adjusted ORs (AORs) and 95% confidence intervals (CIs) were calculated. Data were analysed using SAS (version 9.4, 2013; SAS Institute, Cary, NC, USA). Differences of *CDKN2B‐AS1* expression between PCa cell lines were analysed using Student's *t*‐test in GraphPad Prism 5 (GraphPad Software, San Diego, CA, USA). Statistical significance was set at *p* < 0.05.

## 3 Results

### 3.1 Patient Demographics

Table 1 presents the clinicodemographic characteristics of patients with PCa underwent robot‐assisted RP. The patient cohort predominantly comprised older individuals, with 57.4% of the patients aged > 65 years; the age distribution is consistent with that in previous studies where approximately 60% of all patients with PCa were aged > 65 years [28]. Most patients had early‐stage tumours (clinical stage T1 or T2, 86%), with 60.1% in Gleason grade groups 1 or 2, and the majority showed no lymph node metastasis (pathological N0, 91.4%), lymphovascular invasion (84%) or seminal vesicle invasion (78.6%). Regarding the D'Amico risk classification, approximately 50.4% of the patients were classified as high‐risk patients, indicating a > 50% risk of BCR within 5 years after treatment.

**TABLE 1:** Clinicodemographic characteristics of 695 patients with prostate cancer.

Variables	Patients (*N* = 695) *n* (%)
Age at diagnosis (years)	
≤ 65	296 (42.6)
> 65	399 (57.4)
PSA at diagnosis (ng/mL)	
≤ 7	185 (26.6)
> 7	510 (73.4)
Pathological Gleason grade group	
1 + 2	418 (60.1)
3 + 4 + 5	277 (39.9)
Clinical T stage	
1 + 2	598 (86.0)
3 + 4	97 (14.0)
Clinical N stage	
N0	681 (98.0)
N1	14 (2.0)
Pathological T stage	
2	368 (52.9)
3 + 4	327 (47.1)
Pathological N stage	
N0	635 (91.4)
N1	60 (8.6)
Seminal vesicle invasion	
No	546 (78.6)
Yes	149 (21.4)
Lymphovascular invasion	
No	584 (84.0)
Yes	111 (16.0)
Biochemical recurrence	
No	474 (68.2)
Yes	221 (31.8)
D'Amico classification	
Low risk/intermediate risk	345 (49.6)
High risk	350 (50.4)

Abbreviations: N, node; PSA, prostate‐specific antigen; T, tumour.

### 3.2 Effect of *CDKN2B‐* *AS1* SNPs on PCa Risk

We first explored the correlations of the five selected SNPs (rs564398 [T/C], rs1333048 [A/C], rs1537373 [G/T], rs2151280 [A/G] and rs8181047 [G/A]) of *CDKN2B‐AS1* with the incidence of PCa. As shown in Table 2, the predominant genotypes in healthy controls and patients with PCa for the *CDKN2B‐AS1* SNPs rs564398 and rs8181047 were homozygous T/T and G/G, respectively. Conversely, the most common genotypes for the rs1333048, rs1537373 and rs2151280 SNPs were heterozygous A/C, G/T and A/G, respectively. We calculated AORs with 95% CIs by using multivariate logistic regression models adjusted for age and thus investigated the associations of *CDKN2B‐AS1* SNPs with PCa incidence. Our findings revealed no significant association between *CDKN2B‐AS1* SNPs and PCa incidence. These findings were similar in both dominant and codominant models (Table 2).

**TABLE 2:** Adjusted odds ratios (AORs) and 95% confidence intervals (CIs) for prostate cancer risk in relation to *CDKN2B‐AS1* genotype frequencies.

Variables	Controls (*N* = 695) *n* (%)	Patients (*N* = 695) *n* (%)	AOR (95% CI)	*p*
rs564398				
TT	559 (80.4)	542 (78.0)	1.000 (reference)	
TC	129 (18.6)	145 (20.9)	1.251 (0.940–1.665)	0.125
CC	7 (1.0)	8 (1.1)	1.692 (0.581–4.924)	0.335
TC + CC	136 (19.6)	153 (22.0)	1.271 (0.961–1.682)	0.093
rs1333048				
AA	191 (27.5)	185 (26.6)	1.000 (reference)	
AC	362 (52.1)	371 (53.4)	1.080 (0.826–1.414)	0.573
CC	142 (20.4)	139 (20.0)	0.998 (0.715–1.395)	0.993
AC + CC	504 (72.5)	510 (73.4)	1.057 (0.818–1.365)	0.671
rs1537373				
GG	285 (41.0)	284 (40.9)	1.000 (reference)	
GT	326 (46.9)	322 (46.3)	0.968 (0.759–1.235)	0.794
TT	84 (12.1)	89 (12.8)	1.041 (0.720–1.504)	0.831
GT + TT	410 (59.0)	411 (59.1)	0.983 (0.780–1.239)	0.884
rs2151280				
AA	293 (42.2)	306 (44.0)	1.000 (reference)	
AG	322 (46.3)	312 (44.9)	0.949 (0.745–1.208)	0.668
GG	80 (11.5)	77 (11.1)	1.002 (0.686–1.464)	0.992
AG + GG	402 (57.8)	389 (56.0)	0.959 (0.762–1.206)	0.721
rs8181047				
GG	539 (77.6)	528 (76.0)	1.000 (reference)	
GA	142 (20.4)	156 (22.4)	1.143 (0.866–1.509)	0.344
AA	14 (2.0)	11 (1.6)	1.097 (0.472–2.546)	0.830
GA + AA	156 (22.4)	167 (24.0)	1.140 (0.871–1.491)	0.341

*Note:* AOR: adjusted odds ratio with their 95% CIs were estimated by multiple logistic regression models after controlling for age.

### 3.3 Correlation Between the *CDKN2B‐* *AS1* SNP rs1333048 and Pathological Gleason Grade in Patients With PCa

We analysed the correlations of the five *CDKN2B‐AS1* SNPs with various clinicopathological features of PCa—pathological Gleason grade, T stage and N stage; clinical T stage; tumour invasion status; and D'Amico classification. The patient cohort was further divided into two groups: patients with homozygous wild‐type (WT) alleles and those with at least one polymorphic allele. Notably, patients carrying at least one minor allele (AC or CC) of rs1333048 exhibited a significantly higher risk of developing tumours with high Gleason grades (3 + 4 + 5) (OR: 1.493; 95% CI: 1.049–2.126; *p* = 0.026) than did those with WT homozygotes (AA; Table 3). No significant association was observed between the other four *CDKN2B‐AS1* SNPs (rs564398, rs1537373, rs2151280 and rs8181047) and the clinicopathological features of PCa (Tables 4 and 5). We further stratified patients with PCa into two subgroups by BCR and investigated the correlations of the *CDKN2B‐AS1* SNPs with the clinicopathological features of PCa in each subgroup. A total of 474 patients did not exhibit BCR. Among them, patients carrying the rs1333048 C allele had a significantly higher risk of developing tumours with high Gleason grades (OR: 1.786; 95% CI: 1.083–2.945; *p* = 0.022; Table 6), a trend that was not observed in patients with BCR (data not shown).

**TABLE 3:** Correlation between the clinical status of patients with prostate cancer and the frequency of the *CDKN2B‐AS1* variant rs1333048.

Variables	Genotypic frequencies			
rs1333048	AA (*N* = 185) *n* (%)	AC + CC (*N* = 510) *n* (%)	OR (95% CI)	*p*
Pathological Gleason grade group				
1 + 2	124 (67.0%)	294 (57.6%)	1.000	**0.026** *
3 + 4 + 5	61 (33.0%)	216 (42.4%)	**1.493 (1.049–2.126)**	
Clinical T stage				
1 + 2	163 (88.1%)	435 (85.3%)	1.000	0.344
3 + 4	22 (11.9%)	75 (14.7%)	1.277 (0.769**–**2.123)	
Pathological T stage				
2	101 (54.6%)	267 (52.4%)	1.000	0.601
3 + 4	84 (45.4%)	243 (47.6%)	1.094 (0.781**–**1.534)	
Pathological N stage				
N0	169 (91.4%)	466 (91.4%)	1.000	0.993
N1	16 (8.6%)	44 (8.6%)	0.997 (0.548**–**1.815)	
Seminal vesicle invasion				
No	147 (79.5%)	399 (78.2%)	1.000	0.782
Yes	38 (20.5%)	111 (21.8%)	1.076 (0.711**–**1.628)	
Lymphovascular invasion				
No	155 (83.8%)	429 (84.1%)	1.000	0.915
Yes	30 (16.2%)	81 (15.9%)	0.976 (0.617**–**1.541)	
Biochemical recurrence				
No	128 (69.2%)	346 (67.8%)	1.000	0.736
Yes	57 (30.8%)	164 (32.2%)	1.064 (0.740**–**1.531)	
D'Amico classification				
Low risk/intermediate risk	85 (45.9%)	260 (51.0%)	1.000	0.241
High risk	100 (54.1%)	250 (49.0%)	0.817 (0.583**–**1.145)	

*Note:* Odds ratios and 95% CIs were evaluated using logistic regression models.

Abbreviations: N, node; PSA, prostate‐specific antigen; T, tumour.

* Boldfaced values indicate significance at *p* < 0.05.

**TABLE 4:** Correlations between the clinical status of patients with prostate cancer and the frequencies of the *CDKN2B‐AS1* variants rs564398 and rs1537373.

Variables	rs564398				rs1537373			
	TT (*N* = 542) *n* (%)	TC + CC (*N* = 153) *n* (%)	OR (95% CI)	*p*	GG (*N* = 284) *n* (%)	GT + TT (*N* = 411) *n* (%)	OR (95% CI)	*p*
Pathological Gleason grade group								
1 + 2	319 (58.9)	99 (64.7)	1.000	0.192	165 (58.1)	253 (61.6)	1.000	0.360
3 + 4 + 5	223 (41.1)	54 (35.3)	0.780 (0.537–1.133)		119 (41.9)	158 (38.4)	0.866 (0.636–1.179)	
Clinical T stage								
1 + 2	462 (85.2)	136 (77.1)	1.000	0.250	245 (86.3)	353 (85.9)	1.000	0.887
3 + 4	80 (14.8)	17 (22.9)	0.722 (0.413–1.260)		39 (13.7)	58 (14.1)	1.032 (0.667–1.598)	
Pathological T stage								
2	279 (51.5)	89 (58.2)	1.000	0.143	146 (51.4)	222 (54.0)	1.000	0.499
3 + 4	263 (48.5)	64 (41.8)	0.763 (0.531–1.096)		138 (48.6)	189 (46.0)	0.901 (0.665–1.219)	
Pathological N stage								
N0	494 (91.1)	141 (92.2)	1.000	0.694	259 (91.2)	376 (91.5)	1.000	0.895
N1	48 (8.9)	12 (7.8)	0.876 (0.453–1.694)		25 (8.8)	35 (8.5)	0.964 (0.564–1.650)	
Seminal vesicle invasion								
No	420 (77.5)	126 (82.4)	1.000	0.196	218 (76.8)	328 (79.8)	1.000	0.336
Yes	122 (22.5)	27 (17.6)	0.738 (0.465–1.171)		66 (23.2)	83 (20.2)	0.836 (0.580–1.205)	
Lymphovascular invasion								
No	454 (83.8)	130 (85.0)	1.000	0.720	241 (84.9)	343 (83.5)	1.000	0.619
Yes	88 (16.2)	23 (15.0)	0.913 (0.554–1.503)		43 (15.1)	68 (16.5)	1.111 (0.733–1.684)	
Biochemical recurrence								
No	366 (67.5)	108 (70.6)	1.00	0.473	187 (65.8)	287 (69.8)	1.00	0.267
Yes	176 (32.5)	45 (29.4)	0.866 (0.586–1.282)		97 (34.2)	124 (30.2)	0.833 (0.603–1.151)	
D'Amico classification								
Low risk/intermediate risk	261 (48.2)	84 (54.9)	1.000	0.140	137 (48.2)	208 (50.6)	1.000	0.539
High risk	281 (51.8)	69 (45.1)	0.763 (0.532–1.094)		147 (51.8)	203 (49.4)	0.910 (0.672–1.231)	

*Note:* Odds ratios and 95% CIs were evaluated using logistic regression models.

Abbreviations: N, node; PSA, prostate‐specific antigen; T, tumour.

**TABLE 5:** Correlations between the clinical status of patients with prostate cancer and the frequencies of the *CDKN2B‐AS1* variants rs2151280 and rs8181047.

Variables	rs2151280				rs8181047			
	AA (*N* = 306) *n* (%)	AG + GG (*N* = 389) *n* (%)	OR (95% CI)	*p*	GG (*N* = 528) *n* (%)	GA + AA (*N* = 167) *n* (%)	OR (95% CI)	*p*
Pathological Gleason grade group								
1 + 2	176 (57.5)	242 (62.2)	1.000	0.210	311 (58.9)	107 (64.1)	1.000	0.234
3 + 4 + 5	130 (42.5)	147 (37.8)	0.822 (0.606–1.116)		217 (41.1)	60 (35.9)	0.804 (0.560–1.153)	
Clinical T stage								
1 + 2	260 (85.0)	338 (86.9)	1.000	0.468	448 (84.8)	150 (89.8)	1.000	0.106
3 + 4	46 (15.0)	51 (13.1)	0.853 (0.555–1.311)		80 (15.2)	17 (10.2)	0.635 (0.364–1.106)	
Pathological T stage								
2	162 (52.9)	206 (53.0)	1.000	0.997	274 (51.9)	94 (56.3)	1.000	0.321
3 + 4	144 (47.1)	183 (47.0)	0.999 (0.740–1.349)		254 (48.1)	73 (43.7)	0.838 (0.590–1.189)	
Pathological N stage								
N0	275 (89.9)	360 (92.5)	1.000	0.212	480 (90.9)	155 (92.8)	1.000	0.445
N1	31 (10.1)	29 (7.5)	0.715 (0.421–1.214)		48 (9.1)	12 (7.2)	0.774 (0.401–1.495)	
Seminal vesicle invasion								
No	241 (78.8)	305 (78.4)	1.000	0.911	407 (77.1)	139 (83.2)	1.000	0.091
Yes	65 (21.2)	84 (21.6)	1.021 (0.709–1.471)		121 (22.9)	28 (16.8)	0.678 (0.430–1.067)	
Lymphovascular invasion								
No	252 (82.4)	332 (85.3)	1.000	0.285	443 (83.9)	141 (84.4)	1.000	0.871
Yes	54 (17.6)	57 (14.7)	0.801 (0.534–1.203)		85 (16.1)	26 (15.6)	0.961 (0.596–1.551)	
Biochemical recurrence								
No	213 (69.6)	261 (67.1)	1.00	0.480	359 (68.0)	115 (68.9)	1.00	0.833
Yes	93 (30.4)	128 (32.9)	1.123 (0.814–1.551)		169 (32.0)	52 (31.1)	0.961 (0.660–1.398)	
D'Amico classification								
Low risk/intermediate risk	149 (48.7)	196 (50.4)	1.000	0.658	251 (47.5)	94 (56.3)	1.000	0.076
High risk	157 (51.3)	193 (49.6)	0.935 (0.693–1.261)		277 (52.2)	73 (43.7)	0.704 (0.496–1.051)	

*Note:* Odds ratios and 95% CIs were evaluated using logistic regression models.

Abbreviations: N, node; PSA, prostate‐specific antigen; T, tumour.

**TABLE 6:** Correlation between clinical status and the frequency of the CDKN2B‐AS1 variant rs1333048 in prostate cancer patients without biochemical recurrence.

Variables	Genotypic frequencies			
rs1333048	AA (*N* = 128) *n* (%)	AC + CC (*N* = 346) *n* (%)	OR (95% CI)	*p*
Pathological Gleason grade group				
1 + 2	104 (81.3)	245 (70.8)	1.000	**0.022** *
3 + 4 + 5	24 (18.8)	101 (29.2)	**1.786 (1.083–2.945)**	
Clinical T stage				
1 + 2	116 (90.6)	316 (91.3)	1.000	0.811
3 + 4	12 (9.4)	30 (8.7)	0.918 (0.455**–**1.853)	
Pathological T stage				
2	84 (65.6)	233 (67.3)	1.000	0.725
3 + 4	44 (34.4)	113 (32.7)	0.926 (0.603**–**1.421)	
Pathological N stage				
N0	126 (98.4)	336 (97.1)	1.000	0.414
N1	2 (1.6)	10 (2.9)	1.875 (0.405**–**8.676)	
Seminal vesicle invasion				
No	115 (89.8)	315 (91.0)	1.000	0.690
Yes	13 (10.2)	31 (9.0)	0.871 (0.440**–**1.722)	
Lymphovascular invasion				
No	119 (93.0)	321 (92.8)	1.000	0.942
Yes	9 (7.0)	25 (7.2)	1.030 (0.467**–**2.270)	
D'Amico classification				
Low risk/intermediate risk	67 (52.3)	203 (58.7)	1.000	0.217
High risk	61 (47.7)	143 (41.3)	0.774 (0.515**–**1.163)	

*Note:* Odds ratios and 95% confidence intervals were evaluated using logistic regression models.

Abbreviations: N, node; PSA, prostate‐specific antigen; T, tumour.

* Boldfaced values indicate significance at *p* < 0.05.

### 3.4 Potential Influence of *CDKN2B‐AS1* Genetic Variants on the Expression of *CDKN2B‐AS1*

**FIGURE 1:**
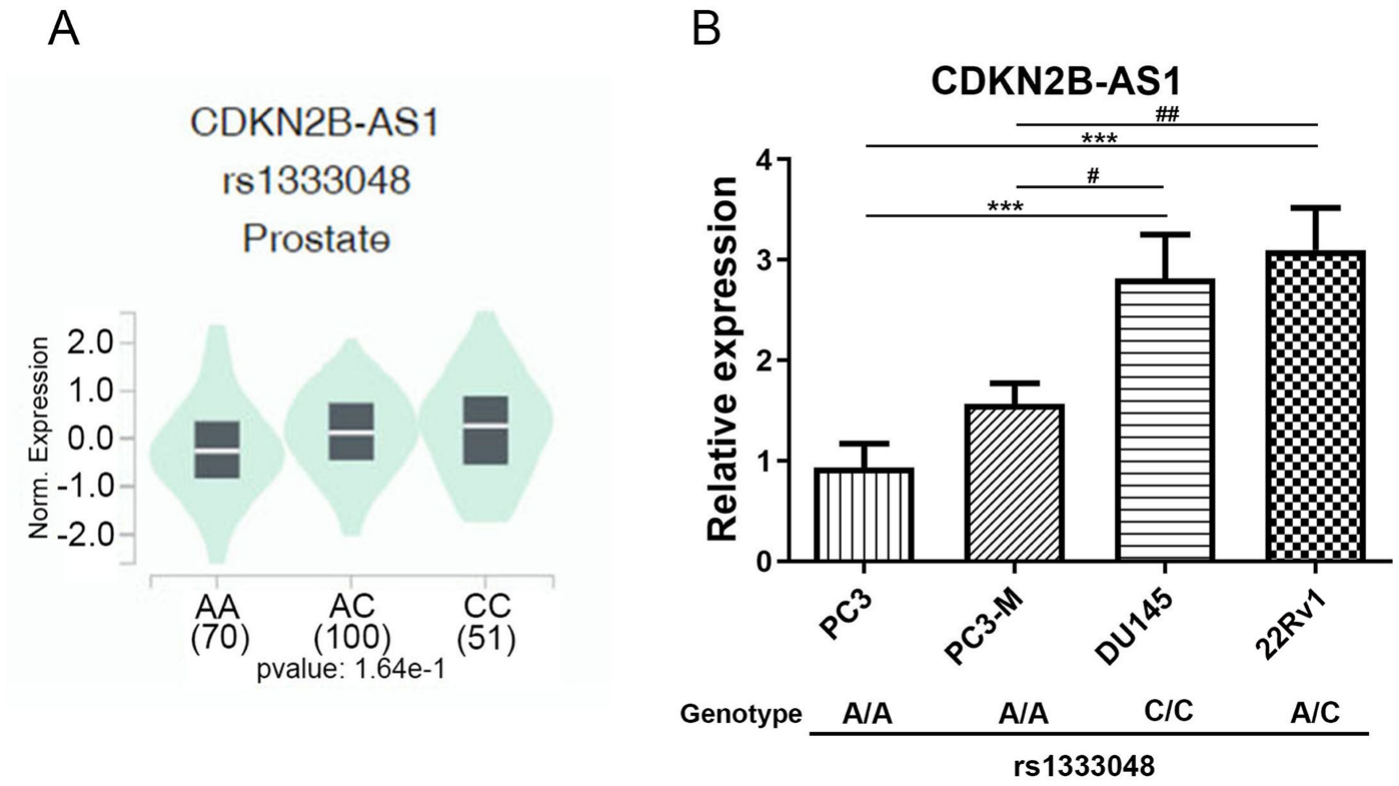
Impacts of CDKN2B‐AS1 rs1333048 polymorphisms on CDKN2B‐AS1 expression. (A) The Genotype Tissue Expression (GTEx) Portal (https://www.gtexportal.org/home/) provided data on CDKN2B‐AS1 expression across different genotypes. The violin plot shows that the C allele of rs1333048 tends to be associated with higher CDKN2B‐AS1 expression levels in prostate tissue. (B) Correlation between CDKN2B‐AS1 rs1333048 genotypes and CDKN2B‐AS1 expression levels was examined in four prostate cancer (PCa) cell lines. In the lower panel, CDKN2B‐AS1 rs1333048 genotypes in PCa cells (PC3, PC3‐M, DU145 and 22Rv1) were identified using a TaqMan SNP Genotyping Assay, while the upper panel shows CDKN2B‐AS1 expression levels determined by RT‐qPCR. Differences are presented as mean ± standard deviation. ***p < 0.001, compared to PC3 cells; # *p* < 0.05, ## *p* < 0.01, compared to PC3‐M cells.

We further investigated the effect of the rs1333048 SNP on *CDKN2B‐AS1* expression by using data from the Genotype‐Tissue Expression (GTEx) database. Our findings revealed a trend, though not statistically significant, towards higher *CDKN2B‐AS1* expression levels in prostate tissues of healthy individuals with the polymorphic C allele of rs1333048 (AC or CC genotype) compared to those with the wild‐type homozygous genotype (AA; Figure 1A). We next examined the correlations between rs1333048 genotypes and *CDKN2B‐AS1* expression levels among four PCa cell lines (PC3, PC3‐M, DU145 and 22Rv1). We observed that 22Rv1 and DU145 cells, respectively, carried the AC and CC genotypes of rs1333048 compared to PC3 and PC3‐M cells, which carried the AA genotype (Figure 1B, lower panel). From the results of RT‐qPCR, we found that 22Rv1 and DU145 cells harbouring at least one minor C allele expressed significantly higher *CDKN2B‐AS1* levels than PC3 and PC3‐M cells harbouring the AA genotype (Figure 1B, upper panel).

### 3.5 Correlations of *CDKN2B‐AS1* Expression Levels With PCa Progression and Prognosis

**FIGURE 2:**
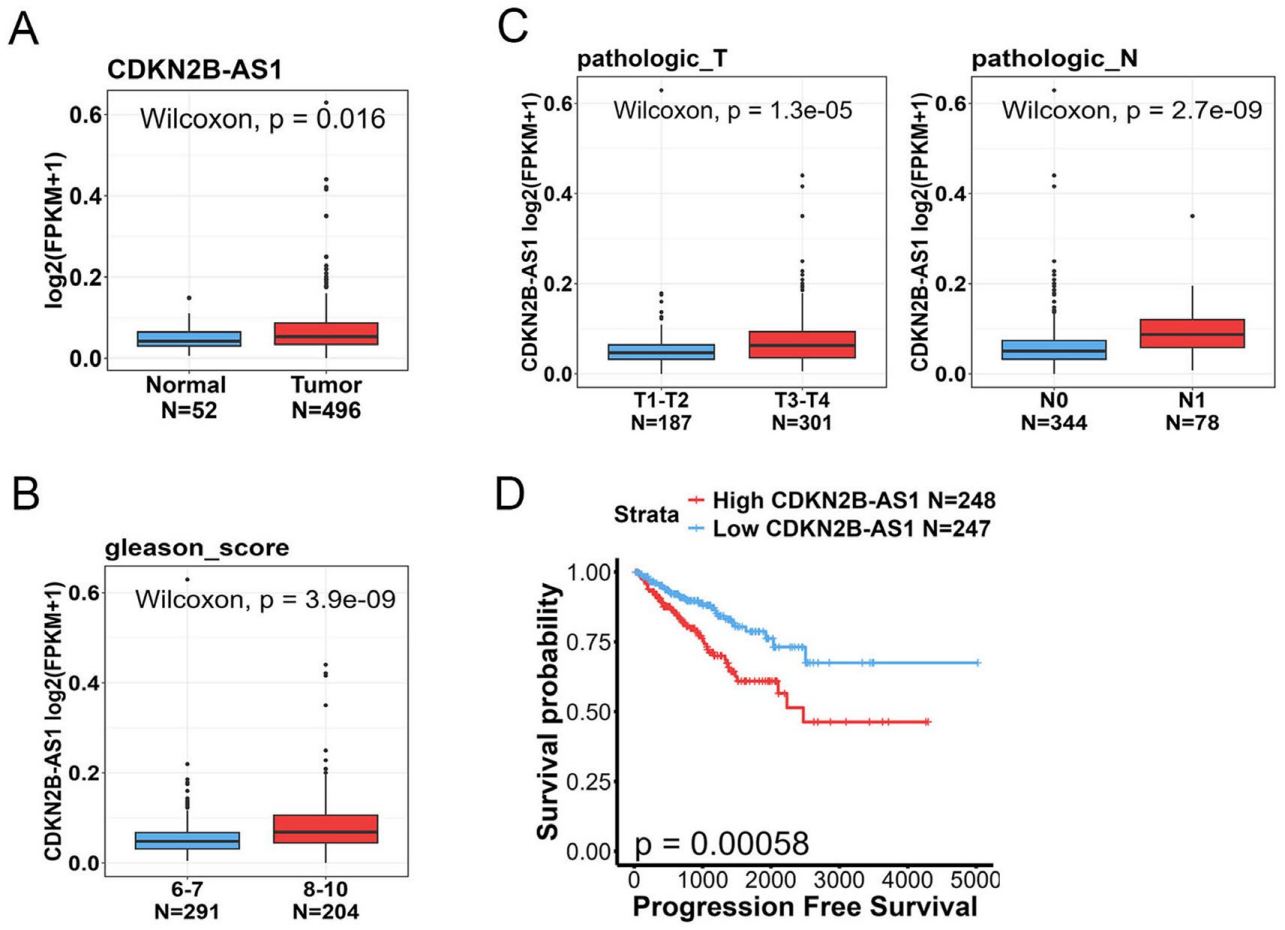
Clinical significance of *CDKN2B‐AS1* expression levels in patients with prostate cancer (PCa). The clinical significance was analysed using data from The Cancer Genome Atlas Prostate Adenocarcinoma dataset. (A) *CDKN2B‐AS1* expression in normal versus PCa tissues. (B and C) Expression levels of *CDKN2B‐AS1* in PCa patients stratified by Gleason scores (B) and pathological T stage and lymph node metastasis (C). (D) Kaplan–Meier curves indicating progression‐free survival in patients with high or low levels of *CDKN2B‐AS1* expression (*CDKN2B‐AS1* ^high^ and *CDKN2B‐AS1* ^low^, respectively). The *p* values indicate the significance of difference between the two expression groups.

To conduct a more thorough analysis of *CDKN2B‐AS1* expression levels in both normal and PCa tissues, as well as to investigate potential correlations between *CDKN2B‐AS1* levels and the progression and prognosis of PCa, we utilised the TCGA‐PRAD dataset. Our findings revealed that the expression level of *CDKN2B‐AS1* was significantly higher in PCa tissues than in normal tissues (Figure 2A). Furthermore, the relative levels of *CDKN2B‐AS1* transcripts were higher in patients with high Gleason scores (Figure 2B), larger tumour sizes and more lymph node metastasis (Figure 2C). A Kaplan–Meier plot indicated that a higher level of *CDKN2B‐AS1* expression was associated with a shorter duration of PFS (Figure 2D).

### 3.6 Analysis of Potential Molecular Mechanisms Governed by *CDKN2B‐AS1* in the Progression of PCa

**FIGURE 3:**
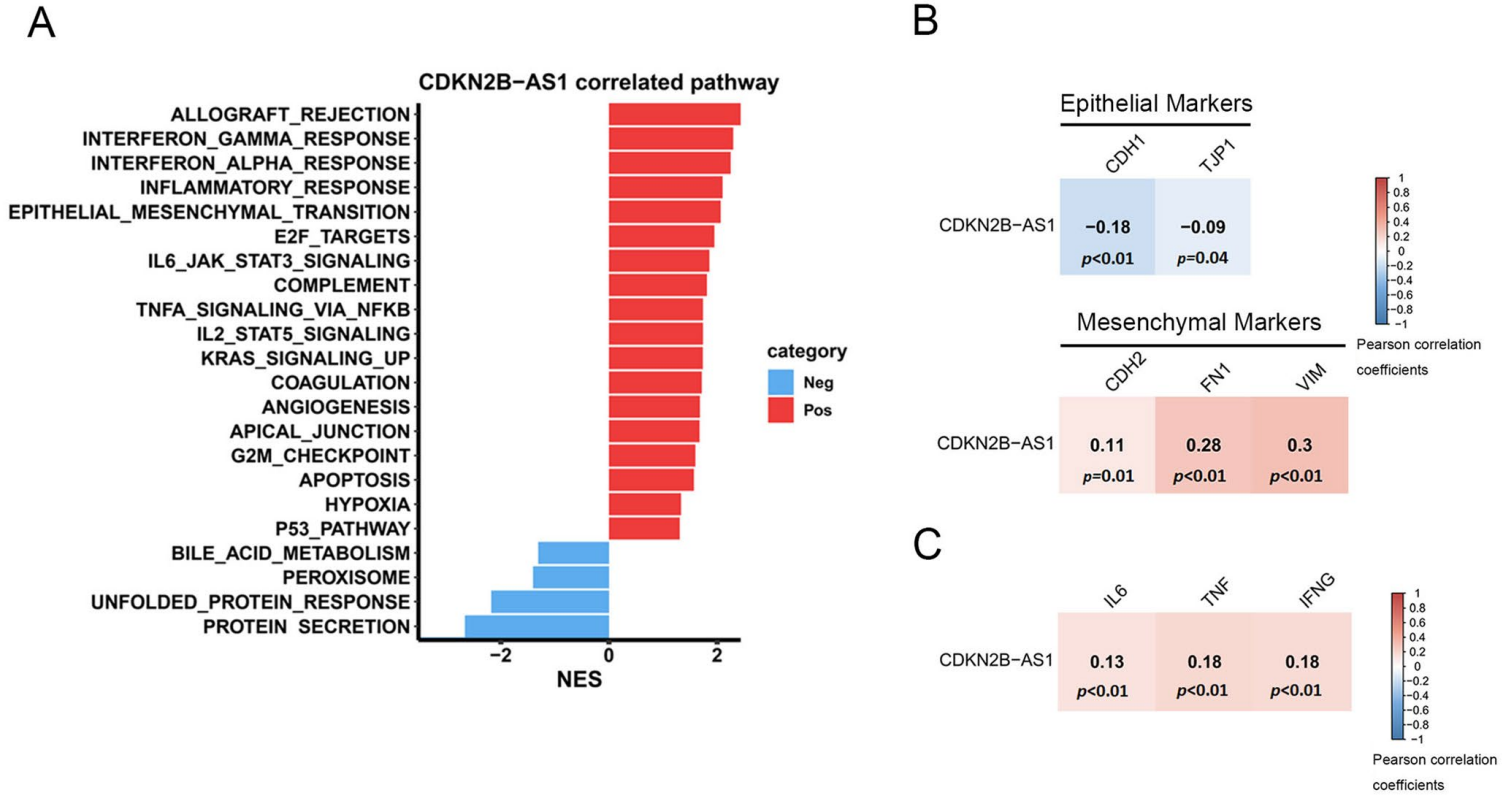
*CDKN2B‐AS1*‐associated pathways in patients with prostate cancer (PCa). (A) A horizontal bar plot indicating the pathways associated with *CDKN2B‐AS1*. Red and blue bars indicate pathways positively and negatively, respectively, associated with *CDKN2B‐AS1*. The x‐axis presents the normalised enrichment scores (NES), whereas the y‐axis presents the pathways identified from the Hallmark database. (B and C) Correlation plots indicating the correlations of *CDKN2B‐AS1* expression with the biomarkers of epithelial–mesenchymal transition (B) and those of inflammatory response (C). RNA sequencing data from The Cancer Genome Atlas Prostate Adenocarcinoma dataset were analysed. Pearson correlation analysis was performed to identify the correlations between *CDKN2B‐AS1* and the aforementioned biomarkers. Correlation coefficients and *p* values are presented in each square, with the scale bar representing the strength of correlation.

To elucidate the mechanisms through which *CDKN2B‐AS1* influences PCa progression, we conducted a GSEA by using TCGA‐PRAD dataset. The analysis revealed several inflammation‐related Hallmark gene sets, such as ‘INTERFERON_GAMMA_RESPONSE’, ‘INTERFERON_ALPHA_RESPONSE’, ‘INFLAMMATORY_RESPONSE’, ‘IL6_JAK_STAT3_SIGNALING’ and ‘TNFA_SIGNALING_VIA_NFKB’ in patients with high levels of *CDKN2B‐AS1* expression (Figure 3A). Evidence suggests that chronic inflammation plays a pivotal role in the development of PCa and its progression to an advanced metastatic disease [29]. Therefore, *CDKN2B‐AS1* may regulate prostatic inflammation to drive the progression of PCa. Additionally, epithelial–mesenchymal transition (EMT) and angiogenesis was found to be associated with *CDKN2B‐AS1* in the context of PCa (Figure 3A). The analysis of human PCa samples from TCGA through the cBioPortal platform revealed that the expression level of *CDKN2B‐AS1* was positively correlated with those of mesenchymal phenotype‐related genes (*CDH2*, *FN1* and *VIM*) and negatively correlated with those of epithelial phenotype‐related genes (*CDH1* and *TJP1*; Figure 3B). As shown in Figure 3C, the expression level of *CDKN2B‐AS1* was also associated with those of inflammation‐related genes (*IFNG*, *IL6* and *TNF*).

## 4 Discussion

PCa is treatable when detected early and monitored closely. However, in advanced stages, tumours may develop resistance to ADT, which leads to its progression to a lethal form [3, 4, 5]. *CDKN2B‐AS1*, an lncRNA first identified in 2011, serves as a prognostic indicator and biomarker of the tumour immune microenvironment in thyroid and endometrial cancers [30, 31]. However, research on the role of *CDKN2B‐AS1* in PCa remains limited. Zhao et al. demonstrated that *CDKN2B‐AS1* promotes the proliferation and migration of PCa cells by targeting the let‐7a/TGF‐β1/Smad pathway [25]. Taheri et al. explored the epidemiological aspects of PCa susceptibility and the clinicopathological features associated with *CDKN2B‐AS1* variants in the Iranian population [32]. Our analysis of TCGA‐PRAD dataset revealed that the expression level of *CDKN2B‐AS1* is higher in Asian individuals than in Caucasian individuals (Figure S1). Given that the Iranian population is classified as a Caucasian population and the Taiwanese population as an Asian population, we hypothesised that *CDKN2B‐AS1* expression would influence PCa outcomes also in the Taiwanese population.

Our study revealed that Taiwanese patients with PCa carrying the mutant C allele of rs1333048 (AC + CC) in *CDKN2B‐AS1*, particularly those without BCR, had a significantly elevated risk of developing tumours with high Gleason grades. This finding is consistent with those of Yeh et al., who indicated that Taiwanese patients with oral cancer carrying at least one minor allele of rs1333048 (AC + CC) were more likely to have tumours with advanced clinical stages and large tumours than were those carrying the major allele (AA) [21]. Additionally, a meta‐analysis by Huang et al. showed that the rs1333048 A/C polymorphism was associated with an increased overall cancer risk across all genetic models [33]. Collectively, these findings suggest that the *CDKN2B‐AS1* SNP rs1333048 A/C can predict the progression of several cancers, including PCa. However, to the best of our knowledge, no correlation has been identified between the rs1333048 SNP and *CDKN2B‐AS1* expression levels in any disease. Recent studies on expression quantitative trait loci (eQTLs) have shown that the expression of most lncRNA genes is affected by genetic variations [34]. We conducted a preliminary analysis of the association between the *CDKN2B‐AS1* variant rs1333048 and *CDKN2B‐AS1* expression levels by using data from the GTEx database. A violin plot corresponding to the eQTLs indicated a trend towards increased *CDKN2B‐AS1* expression in prostate tissues from individuals carrying the polymorphic C allele of rs1333048. Moreover, PCa cells harbouring at least one minor C allele also exhibited significantly higher *CDKN2B‐AS1* levels than those with the AA genotype. In line with previous studies showing the upregulation of *CDKN2B‐AS1* in various solid tumours, such as kidney [35], laryngeal squamous cell [36], ovarian [37], thyroid [30] and liver [38] cancers, elevated *CDKN2B‐AS1* levels have been linked to advanced TNM stages or poor prognoses in these cancer types. Our results similarly demonstrated that *CDKN2B‐AS1* transcripts in PCa were higher compared to noncancerous tissues in the TCGA‐PRAD dataset and were associated with advanced T and N stages, higher Gleason scores and poor prognostic outcomes. Collectively, these findings suggest that the *CDKN2B‐AS1* SNP rs1333048 may influence the expression of *CDKN2B‐AS1*, leading to PCa progression and thus an unfavourable prognosis. However, further studies are needed to confirm the correlation between rs1333048 and serum *CDKN2B‐AS1* levels in patients with PCa.

Mak et al. demonstrated that PCa tumours with higher Gleason grades exhibited significantly higher expression levels of HIF‐1α and vascular endothelial growth factor (VEGF), along with higher levels of nuclear localisation of Snail. The researchers further reported that the difference between high‐ and low‐Gleason grade PCa tumours was attributable to the activation of an EMT dedifferentiation programme driven by the HIF‐1α/VEGF/neuropilin‐1 pathway [39]. The loss of E‐cadherin has been associated with PCa progression and high Gleason grades; this finding indicates the potential of E‐cadherin as a prognostic marker of disease progression [40]. We found that *CDKN2B‐AS1* levels were positively associated with EMT‐related gene signatures in TCGA‐PRAD dataset and also with poor PFS. An analysis of human PCa samples through the cBioPortal platform revealed that the expression level of *CDKN2B‐AS1* was positively correlated with those of mesenchymal phenotype‐related genes and negatively correlated with those of epithelial phenotype‐related genes. Together, these findings suggest that high levels of *CDKN2B‐AS1* expression lead to high Gleason grades and accelerated PCa progression through the promotion of EMT. In addition to its role in EMT, *CDKN2B‐AS1* mediates several inflammation‐related pathways in PCa—for example, the IFN‐γ response, IL‐6/Janus kinase/signal transducer and activator of transcription 3, and TNF‐α/nuclear factor‐κB pathways. An analysis of TCGA dataset revealed significant correlations between the expression level of *CDKN2B‐AS1* and those of *IFNG*, *IL6* and *TNF* in patients with PCa. Evidence suggests that CDKN2B‐AS1 promotes cancer malignancy by modulating immune cell infiltration, as observed in thyroid and endometrial cancers [30, 31]. The downregulation of *CDKN2B‐AS1* expression was demonstrated to suppress the inflammatory response in mice with allergic rhinitis [41]. Genetic variants of *CDKN2B‐AS1*—for example, rs1333048—have been associated with aggressive periodontitis and elevated high‐sensitivity C‐reactive protein levels in patients with periodontitis [42, 43]. C‐reactive protein, a general marker of inflammation, is also associated with poor PFS in patients with PCa [44]. Moreover, chronic inflammation observed in benign prostate biopsy specimens has been linked to the presence of high Gleason grade tumours in nearby regions [45]. Collectively, these findings suggest that the genetic variant rs1333048 may influence *CDKN2B‐AS1* expression, modulate the immune microenvironment and consequently promote PCa progression.

Our study still has certain limitations that should be noted. First, while all PCa patients in this SNP study were Taiwanese (of Asia ethnicity), the correlations between *CDKN2B‐AS1* expression levels and clinicopathological features or prognosis were analysed using the TCGA‐PRAD dataset, which predominantly includes Caucasian and African American individuals. Although Asian individuals showed a trend towards higher *CDKN2B‐AS1* expression in PCa tissues compared to Caucasian and African American individuals (Figure S1), further studies are needed to validate the correlation between *CDKN2B‐AS1* expression and clinicopathological features specifically in Taiwanese PCa tissues. Additionally, whether the impact of *CDKN2B‐AS1* rs1333048 SNPs on PCa development is consistent across different racial/ethnic groups remains unclear, underscoring the need for studies with larger, independent cohorts from diverse medical centres worldwide to confirm our findings. Finally, our study only indicated the potential effects of *CDKN2B‐AS1* rs1333048 SNPs on *CDKN2B‐AS1* expression in prostate tissues of healthy individuals based on the GTEx database or PCa cell lines. Future research should collect both mRNA and DNA from the same PCa patient samples to confirm the influence of *CDKN2B‐AS1* SNPs on *CDKN2B‐AS1* expression in PCa patients.

To the best of our knowledge, this study is the first to investigate the associations of *CDKN2B‐AS1* variants with the clinicopathological features of PCa in the Taiwanese population. Our findings indicated that *CDKN2B‐AS1*‐associated pathways, such as EMT and inflammatory response, can serve as potential drivers of PCa progression. Furthermore, the *CDKN2B‐AS1* variant rs1333048 may serve as valuable biomarker of tumour aggressiveness and prognosis in PCa.

## Author Contributions

**Min‐Che Tung:** conceptualization (equal), data curation (equal), methodology (equal), writing – original draft (equal). **Chia‐Yen Lin:** data curation (equal), resources (equal). **Yu‐Ching Wen:** data curation (equal), funding acquisition (equal). **Lun‐Ching Chang:** software (equal). **Shun‐Fa Yang:** conceptualization (equal), methodology (equal), writing – original draft (equal). **Ming‐Hsien Chien:** conceptualization (equal), funding acquisition (equal), software (equal), writing – original draft (equal), writing – review and editing (equal).

## Conflicts of Interest

The authors declare no conflicts of interest.

## Supporting information


Figure S1.

## Data Availability

The data used to support the findings of this study are available from the corresponding author upon request.
