# Supplementary material for: Associations of the Expression Levels and Risk Variants of CDKN2B‐AS1 Long Noncoding RNA With the Susceptibility and Progression of Prostate Cancer
Source: J Cell Mol Med. 2024 Dec 4;28(23):e70264. doi: 10.1111/jcmm.70264 (PMC11617473; doi:10.1111/jcmm.70264)
Supplement: Supplementary file 1 — Figure S1. [file JCMM-28-e70264-s001.docx]

**Supplementary information**

**Title:**

**Associations of** **the expression levels and risk variants of *CDKN2B-AS1* long noncoding RNA with the susceptibility and progression of prostate cancer**

Min-Che Tung, Chia-Yen Lin, Yu-Ching Wen, Lun-Ching Chang, Shun-Fa Yang*, and Ming-Hsien Chien* (E-mail: ysf@csmu.edu.tw and mhchien1976@gmail.com)

**Figure Legends**

**
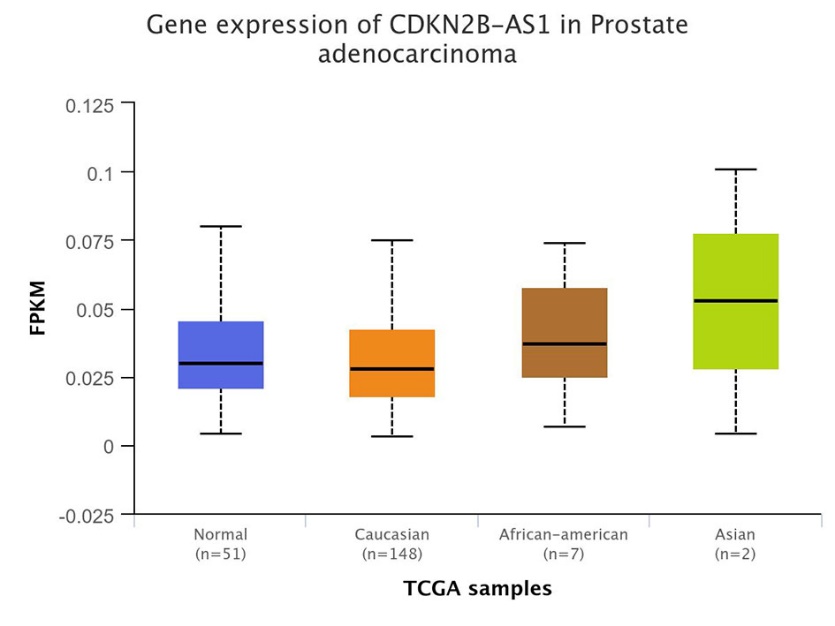
**

**Supplementary Figure 1.** Expression level of *CDKN2B-AS1* in normal versus prostate cancer tissues across races. The comparison was performed using data from the University of Alabama Cancer Database.
